# Prevalence, antimicrobial susceptibility and risk factors associated with non-typhoidal *Salmonella* on Ugandan layer hen farms

**DOI:** 10.1186/s12917-017-1291-1

**Published:** 2017-11-29

**Authors:** Terence Odoch, Yngvild Wasteson, Trine L’Abée-Lund, Adrian Muwonge, Clovice Kankya, Luke Nyakarahuka, Sarah Tegule, Eystein Skjerve

**Affiliations:** 1 Department of Bio-security, Ecosystems and Veterinary Public Health, College of Veterinary Medicine, Animal Resources and Biosecurity (COVAB), Makerere University, Kampala, Uganda; 2 Department of Food Safety and Infection Biology, Faculty of Veterinary Medicine, Norwegian University of Life Sciences (NMBU), Oslo, Norway; 3 Divisions of Genetics and Genomics, Roslin Institute, The Royal (Dick) Vet School of Veterinary Studies, University of Edinburgh, Edinburgh, UK

**Keywords:** Non-typhoidal *Salmonella*, Antimicrobial susceptibility, Risk factor, Layer hens, Prevalence

## Abstract

**Background:**

Non-typhoidal *Salmonella* (NTS) are among the leading global foodborne pathogens and a significant public health threat. Their occurrence in animal reservoirs and their susceptibilities to commonly used antimicrobials are poorly understood in developing countries. The aim of this study was to estimate the prevalence, determine antimicrobial susceptibility and identify risk factors associated with NTS presence in laying hen farms in Uganda through a cross-sectional study.

**Results:**

Pooled faecal samples were collected from 237 laying hen farms and these were analysed for NTS following standard laboratory procedures. In total, 49 farms (20.7%; 95% Confidence interval (CI): 15.6–25.6%) were positive for NTS presence. Altogether, ten *Salmonella* serotypes were identified among the confirmed 78 isolates, and the predominant serotypes were *Salmonella* Newport (30.8%), *S*. Hadar (14.1%), *S*. Aberdeen (12.8%), *S*. Heidelberg (12.8%), and *S*. Bolton (12.8%). Phenotypic antimicrobial resistance was detected in 45(57.7%) of the isolates and the highest resistance was against ciprofloxacin (50.0%) followed by sulphonamides (26.9%) and sulphamethoxazole/trimethoprim (7.7%). Resistance was significantly associated with sampled districts (*p* = 0.034). Resistance to three or more drugs, multi-drug resistance (MDR) was detected in 12 (15.4%) of the isolates, 9 (75%) of these were from Wakiso district. A multivariable logistic model identified large farm size (OR = 7.0; 95% CI: 2.5–19.8) and the presence of other animal species on the farm (OR = 5.9; 95% CI: 2.1–16.1) as risk factors for NTS prevalence on farms. Having a separate house for birds newly brought to the farms was found to be protective (OR = 0,4; 95% CI: 0.2–0.8).

**Conclusion:**

This study has highlighted a high prevalence and diversity of NTS species in laying hen farms in Uganda and identified associated risk factors. In addition, it has demonstrated high levels of antimicrobial resistance in isolates of NTS. This could be because of overuse or misuse of antimicrobials in poultry production. Also importantly, the insights provided in this study justifies a strong case for strengthening One Health practices and this will contribute to the development of NTS control strategies at local, national and international levels.

**Electronic supplementary material:**

The online version of this article (10.1186/s12917-017-1291-1) contains supplementary material, which is available to authorized users.

## Background

Non-typhoid *Salmonella* (NTS) is one of the leading bacterial causes of food-borne illnesses, posing huge challenges to public health systems around the world [1–3]. A recent estimate of the global burden of NTS morbidity and mortality showed that enteric NTS cause 93.8 million illnesses with 155,000 deaths annually, while invasive NTS were estimated to cause 3.4 million cases with 681,316 deaths annually [4–6]. African countries have a relatively low level of reported NTS gastroenteritis, but a much higher level of invasive non-enteric NTS infections, estimated at 227 cases per 100,000 persons per year compared to the global average of 49 cases per 100,000 persons per year [4]. This distribution of salmonellosis makes Africa the leading continent with invasive non-enteric NTS cases accounting for more than half of the global cases [4]. In humans, many of the gastroenteric infections caused by NTS are self-limiting, and thus, many sporadic cases go unnoticed and/or unreported. However, a serious aspect of this situation is that some of the gastroenteric infections may develop into bacteraemia, which is an emerging opportunistic infection in individuals infected with HIV, the elderly and in children [7, 8].

The reservoirs of food-borne NTS are often located in the primary food animal production. Many of these zoonotic NTS are able to colonize the intestinal tract of a variety of animal species, and in most of these cases, the animals are healthy and asymptomatic. Faecally contaminated foodstuffs like meat, eggs, dairy products and sometimes vegetables are the main sources of salmonellosis in humans [2, 3, 9, 10]. The dissemination of NTS is also a growing concern due to increasing cases of drug resistant isolates and their frequent carriage of transmissible antimicrobial resistance genes. Even more worrying is the rising occurrence of multidrug-resistant NTS, including cases reported in some African countries [5, 11]. Because of multidrug-resistance, treatment with first line drugs is often no longer an alternative, and this puts pressure on the use of second or third line drugs. Some limited studies in Africa on antimicrobial resistance in NTS isolates from animal sources have been undertaken, but with varying results [11–13].

Many prevalence and risk factor studies of NTS in layer and broiler populations have been conducted in the USA and Europe [14–17]. A systematic review of risk factors associated with laying hen farms identified multiple risk factors related to biosecurity measures, management factors and the environment [18]. In addition, developed countries have put in place monitoring and surveillance systems for antimicrobial resistance targeting important zoonotic pathogens like NTS. Unfortunately, such systematic surveillance neither exist for NTS nor other food-borne pathogens in most developing countries, including Uganda [19]. Consequently, the role of poultry as a reservoir and source of NTS in developing countries is poorly understood. Furthermore, the development of antibiotic resistance of NTS to commonly used antibiotics in agricultural production needs prompt investigation, commencing with susceptibility testing. The significant data gaps in developing countries hinder development of effective control systems and risk-mitigation strategies at multiple levels.

Since most human NTS infections originate from animal sources, prevention and control at pre-harvest level in the primary production units is an effective way to minimize NTS dissemination and transmission to humans through the food chains [20–22]. The aim of this study was to estimate the prevalence, test antimicrobial susceptibility and identify risk factors of NTS using production and management information from a sample of commercial laying chicken farms.

## Methods

### Study area and recruitment of farms

The study was carried out between June 2015 and August 2016 in the districts of Wakiso, Masaka and Lira in Uganda. Wakiso district (00°24′N, 32° 29′E) and Masaka district (00° 30′S, 31° 45′E) are located in the central region of Uganda, while Lira district is located in northern region of Uganda (02°20′N, 33°06′E). These districts were purposively selected because of their high commercial poultry households that make them the hub of poultry industry in Uganda. The sampling frame was generated from a list of farmers participating in National Agricultural Advisory Services (NAADS) program in the three selected districts of Uganda. In Uganda, the majority of poultry farmers are smallholders, and therefore the inclusion criterion was that for a farm to participate, it should have a minimum number of 50 chicken. A sample size calculator, FreeCalc sample size calculation for imperfect tests (www.epitools.ausvet.com.au, accessed on 3rd, June, 2015) was used. The required sample size for demonstrating disease freedom was calculated as previously described [23, 24]. The calculator had the following input; population size (Masaka = 147, Lira = 145, Wakiso = 77), sensitivity (60%), specificity (100%), design prevalence (5%), and the desired type 1 and type 2 errors were all assumed at 0.05. Because of small populations, the modified hypergeometric option was used. A total sample size of 237 (Masaka = 85, Lira = 84, Wakiso = 68) was calculated for individual districts. Computer-generated random numbers were used to select farms. After selection, farmers (respondents) were asked for their cooperation and willingness to participate, and after that a verbal consent was obtained. Those who were not willing to participate were replaced by random selection of others from the same list. Questionnaires were administered to all participating farms and faecal samples were taken for bacteriological analysis.

### Sample collection

Flocks and poultry house sizes varied a lot in the study areas; and because of this, the sampling scheme was standardized. In cases where a farm had more than one poultry house, but with the same age group of birds in each house, one house was randomly selected and sampled. On the other hand, if a farm had more than one house but with different age groups, all the houses were sampled. Each house was divided into sectors of about 5 m by 5 m (25 m^2^), an approach adapted from previous studies [25, 26]. One sample was collected from each sector using sterile gloves and boot swabs. The samples from each house were pooled together, transferred to a properly labelled sterile container and put in a cool box with ice packs. The samples were transported to the laboratory within less than 8 h and processing began immediately. All flocks were sampled once.

### Bacterial diagnostics and identification of *Salmonella* serotypes

Culture and isolation of *Salmonella* spp. followed standard procedures according to *ISO 6579:2002/Amd 1:2007 Annex D: Detection of Salmonella spp. in animal faeces and in environmental samples from the primary production* [27]*.* Briefly, pooled samples were homogenized, 25 g weighed and added to 225 ml of Buffered Peptone Water (BPW) for pre-enrichment and incubated for 20 h at 37 °C. The culture obtained was subjected to selective Modified Semisolid Rappaport Vassiliadis (MSRV) agar plates and incubated at 41.5 °C for 24–48 h. One colony from each culture indicative of NTS was further plated on selective Xylose Lysine Deoxycholate (XLD) agar and incubated at 37 °C for 24 h. Presumptive NTS colonies were stored at −20 °C in Mueller-Hinton agar. The samples were later transported to Norway where they were sub-cultured on blood agar plates and stored at 4 °C. Biochemical confirmatory tests were done by using the API-20E (BioMerieux, Marcy l’Etoile, France) identification system. All isolates were serotyped according to the Kauffman–White–Le–Minor technique [28] at the Norwegian Veterinary Institute.

### Antibiotic susceptibility testing

Antibiotic susceptibility testing of all identified isolates was performed on 13 antibiotics (NEO-SENSITABS™, Rosco, Denmark) using the standard Kirby-Bauer disk diffusion methods on Muller-Hinton agar. Interpretation of sensitive (S), intermediate (I) or resistant (R) was done according to Clinical and Laboratory Standards Institute [29], except for ciprofloxacin (CIPR1μg) which was interpreted using CLSI [30]. The 13 antibiotics were selected based on the common antibiotics used in Uganda and those recommended by World Health Organization (WHO) for routine integrated antimicrobial resistance monitoring [31]. These were gentamycin (GEN10μg), sulfonamide (SULFA240μg), trimethoprim-sulfamethoxazole (SxT 25 μg), ciprofloxacin (CIPR1μg), cefotaxime (CTX 30 μg), meropenem (MPR 10 μg), chloramphenicol (CLR30μg), ceftazidime (CAZ30μg), ampicillin (AMP10μg), amoxycillin clavulanic acid (AMC30μg), trimethoprim (TRIM5μg), tetracycline (TET30μg), and enrofloxacin (ENROF10μg). *Escherichia coli* ATCC 25922 was used as quality control. NTS isolates showing resistance to three or more antibiotics were classified as multidrug-resistant.

### Questionnaire administration

All questionnaires were directly administered onsite after pre-testing. Pre-testing of the questionnaire was done by three trained research assistants in neighbouring districts of Kampala and Mukono in central Uganda and Kole in northern Uganda. These districts have similar production and management systems to the study districts. After pre-testing, the research team reviewed the questionnaires before final administration. The final questionnaire had 80% close-ended questions and was used to collect variables to determine risk factors. The questionnaires collected information on general farm management practices and characteristics, disease prevention, control and management as well as demographic data of the farmers and managers. The questionnaire was written in English, but the research assistants would determine whether the respondent was competent in English or not. Where the respondent was not competent in English, the research assistant would translate in the local language of Luganda (in the case of Wakiso and Masaka districts) and Lwo (in the case of Lira district). The selected households were identified with the help of the local veterinary personnel and chairpersons of local council one. Local council one is the smallest administrative unit in Uganda. Data entry was done by a trained assistant at the College of Veterinary Medicine, Animal Resources and Biosecurity, Makerere University, Kampala, Uganda.

### Data management and analysis

After establishing the database, data were exported to the Statistical Package for Social Scientists (SPSS, version 20) computer program for further data analyses. A farm was considered positive if one of the pooled faecal samples taken tested positive for NTS. An initial descriptive statistics using tables and chi-square testing was performed to assess the association of each variable independently. This was followed by a multivariable logistic model built based upon variables with *p*-values < 0.20 in the initial analyses. All candidate variables were tested for collinearity with other variables using tabulation, and if collinearity was found, the most biologically relevant variable was chosen. The model was built utilizing a backward selection among the candidate variables (*p* < 0.20 from initial analyses) strategy using the Likelihood Ratio test with for comparing models [32]. The final model was assessed for fit using the Hosmer-Lemeshow test [33].

## Results

### Number of samples, NTS prevalence and serotypes

A total of 237 farms participated in the study (Wakiso, *n* = 68; Lira, *n* = 84; Masaka, *n* = 85). Sampling according to the standardized sampling scheme resulted in 366 pooled samples from the 237 farms. Of the 237 farms, 49 (20.7%; 95% CI = 15.6,-25.6%) were positive for NTS. Salmonella isolates were recovered from 78 of the 366 samples (21.3%).

Ten *Salmonella* serotypes were identified from the 78 isolates recovered (Table 1). Of the 49 NTS-positive farms, five farms were contaminated with two different serotypes. All these farms had three or more poultry houses and the serotypes were from different houses. The farms were from the two districts of Wakiso and Masaka representing the central part of Uganda.

**Table 1:** Distribution of NTS serotypes in layer hen farms in Uganda

Serotype	Number of farms	Percentage (%) of positive farms	Percentage (%) of farms investigated
*S*. Newport	13	26.5	5.5
*S.* Hadar	7	3.0	3.0
*S*. Aberdeen	10	20.4	4.2
*S.* Heidelberg	7	3.0	3.0
*S.* Bolton	6	6.0	2.5
*S.* Enteritidis	4	8.2	1.7
*S*. Mbandaka	3	6.1	1.3
*S.* Kampala	2	4.1	0.8
*S.* Typhimurium	1	2.0	0.4
*S.* Uganda	1	2.0	0.4

### Antimicrobial susceptibility of NTS

Forty five, 57.7% (95% CI: 47.4–67.9%) of the 78 isolates were resistant to at least one antibiotic in the disc diffusion test. Resistance varied significantly by district (*p* = 0.034); highest in Wakiso with 75.9% of the isolates from the district resistant to at least one of the tested antibiotics; this was followed by Lira with 52.0% and Masaka with 41.7% of the isolates resistant. The highest resistance was against ciprofloxacin (50.0% of the isolates) followed by sulphonamide (26.9%), sulfamethoxazole/trimethoprim (7.7%), trimethoprim (7.7%), and tetracycline (5.1%), then ampicillin (5.1%), chloramphenicol (5.1%), and enrofloxacin (5.1%). All the isolates were susceptible to meropenem, gentamycin, amoxicillin-clavulanic acid, ceftazidime, and cefotaxime. Multidrug-resistance was seen in 12 (15.4%) of the isolates and five multidrug-resistant phenotypes were identified (Table 2).

**Table 2:** Multidrug resistant profiles of NTS isolates from Wakiso, Lira and Masaka districts, Uganda 2017

Serotype	Resistance profile	No of isolates	District where isolates were recovered
*S*. Bolton	CIPR, SULFA, TET	1	Lira
*S*. Mbandaka	CIPR, CLR, AMP	4	Masaka (2), Wakiso (2)
*S*. Hadar	SULFA, TRIM, SxT	4	Wakiso
*S*. Hadar	CIPR, SUL, TRIM, SxT	2	Wakiso
*S*. Newport	CIPR, SULFA, TET, ENROF	1	Wakiso

*AMP* Ampicillin, *CIPR* Ciprofloxacin, *CLR* Chloramphenicol, *ENROF* Enrofloxacin, *SULFA* Sulphonamides, *SxT* Sulfamethoxazole-trimethoprim, *TET* Tetracycline, *TRIM* Trimethoprim

### Factors associated with NTS

Some key demographic factors, farm management, disease prevention and control practices showing significant association with the prevalence of NTS on farms are presented in Table 3. These were included as candidate variables for the multivariable logistic model (insert Table 3).

**Table 3:** Key demographic factors, disease management practices and farm characteristics associated with the prevalence of *Salmonella*, with *p*-values from simple chi-square analyses

Variable	Category	*Salmonella* positive farms (%)	*p*-value
Sex of farmer	Male (*n* = 108)	31 (28.7)	0.004
	Female (*n* = 129)	18 (14.0)	
Sex of manager	Male (*n* = 85)	27 (31.89)	< 0.001
	Female (*n* = 115)	12 (10.4)	
	Not applicable (*n* = 37)		
Age of the manager	< 20 years (*n* = 5)	4 (80.0)	0.001
	20–35 years (*n* = 103)	18 (17.5)	
	36–50 years (*n* = 85)	23 (27.1)	
	> 50 years (*n* = 35)	3 (8.6)	
	Missing (*n* = 9)		
Education level of the farmer	Primary (*n* = 44)	5 (11.4)	0.012
	Secondary (*n* = 76)	12 (15.8)	
	Tertiary (*n* = 102)	31 (30.4)	
	Missing (*n* = 15)		
Farm size (no. of birds)	Small (50–500) (*n* = 162)	19 (11.7)	< 0.001
	Medium (501–1000) (*n* = 33)	14 (42.4)	
	Large (> 1000) (*n* = 38)	14 (36.8)	
	Missing (*n* = 4)		
Number of poultry houses	One house (*n* = 135)	12 (8.9)	< 0.001
	Two houses (*n* = 45)	8 (17.8)	
	Three houses (*n* = 32)	21 (65.6)	
	> 3 houses (*n* = 25)	8 (32.0)	
Management system	Free range (*n* = 47)	2 (4.3)	< 0.001
	Semi intensive (*n* = 90)	11 (12.2)	
	Intensive (*n* = 98)	35 (35.7)	
	Others (*n* = 2)		
Use of protective clothing	Yes (*n* = 136)	35 (25.7)	0.031
	No (*n* = 99)	14 (14.1)	
	Missing (*n* = 2)		
Who does vaccination	Private (*n* = 88)	19 (21.6)	0.029
	Self/family (*n* = 136)	25 (18.4)	
	Employee (*n* = 9)	5 (55.6)	
	Missing (*n* = 4)		
Reuse of egg trays	Yes (*n* = 105)	29 (27.6)	0.034
	No (*n* = 119)	19 (16.0)	
	Missing (*n* = 13)		
Who treats the birds	Self (*n* = 155)	22 (14.2)	< 0.001
	Government/Animal Health Worker (*n* = 11)	0 (0.0)	
	Private/Animal Health worker (*n* = 52)	24 (46.2)	
	Missing (*n* = 19)		
Presence of other livestock	Present (*n* = 101)	26 (25.7)	0.097
	Not present (*n* = 136)	23 (16.9)	
Having a separate house for new birds	Yes (*n* = 136)	24 (17.6)	0.134
	No (*n* = 97)	25 (25.8)	
	Missing (*n* = 4)		
Disposal of dead birds	Burying (*n* = 109)	21 (19.3)	< 0.001
	Burning (*n* = 17)	12 (70.6)	
	Throw away (*n* = 45)	9 (20.0)	
	Giving to animals (dogs and pigs) (*n* = 32)	4 (12.5)	
	Drop in a pit (*n* = 24)	3 (12.5)	
	Missing (*n* = 10)		
Keeping of pets	Yes (*n* = 137)	35 (25.5)	0.025
	No (*n* = 99)	14 (14.1)	
	Missing (*n* = 1)		
If yes, species of pets	Dogs (*n* = 62)	20 (32.3)	0.006
	Cats (*n* = 24)	0 (0.0)	
	Both dogs and cats (*n* = 51)	15 (29.4)	
Keeping of records	Yes (*n* = 153)	43 (28.1)	< 0.001
	No (*n* = 80)	5 (6.2)	

A final multivariable logistic model identified some risk factors for presence of *Salmonella* spp. (Table 4). Due to collinearity between some variables, type of poultry house and number of houses were dropped (collinear with farm size). Having a written biosecurity plan, and having a separate house for sick birds were also dropped (collinear with having a separate house for new chicken). The final multivariate logistic model found that larger farms had significantly more NTS. Similarly, presence of other livestock species like cattle, goats, sheep, pigs were significantly associated with presence of NTS (Table 4). Another variable that came out to be associated with presence of NTS was keeping of records. On the other hand, the use of separate houses for birds newly brought into the farms reduced the probability for presence of NTS. The model fit was, however, limited, as shown by the Hosmer Lemeshow test (*p* < 0.05).

**Table 4:** Results from multivariable logistic regression showing identified factors associated with *Salmonella* spp. prevalence, with odds ratios with 95% Confidence Interval (CI) and corresponding p-values for the variables

Variable	Level	Odds Ratio	95% CI	*p*-value
Farm size (no. of birds)	Medium vs small	7.0	2.5–19.8	< 0.001
	Large vs small	5.9	2.1–16.1	0.001
Presence of other animal species	Present vs absent	5.0	2.1–16.1	< 0.001
Houses for new birds	Present vs absent	0.4	0.2–0.8	0.014
Records	Present vs absent	6.7	2.2–20.2	0.001

## Discussion

This study represents, to our knowledge, the first estimate of the prevalence of NTS in laying hen farms in Uganda. NTS prevalence was estimated at 21% of the farms with ten different serotypes identified. High phenotypic resistance to antimicrobials was found among the isolates with almost 58% of the isolates found resistant. In this study, the logistic regression model identified large farm size, presence of other poultry, and keeping of records as factors associated with occurrence of NTS in Uganda.

NTS prevalence of 21% is not surprisingly high considering how the poultry industry operates in the country, where disease control efforts are poor and deficient. In Uganda, chickens are not vaccinated against NTS. The available vaccines is for fowl typhoid targeting *Salmonella* Gallinarum and the protocol used is not ideal for identification of *S*. Gallinarum. Vaccinations of poultry in Uganda are not mandatory. Most commercial layer hen farms include fowl typhoid vaccinations in their routine vaccination schedules. A similar study in Senegal reported detection of NTS in faecal samples in 35.1% of farms [34] and in Nigeria, a related study reported isolation of NTS in 12.5% of poultry droppings [35]. A more recent study in Nigeria by Fagbamilla et al. [36] estimated NTS prevalence of 43.6% in commercial poultry farms. In Algeria, a study in laying hen flocks by Bouzidi et al. [37] found that eight out 18 flocks were contaminated with NTS.

The identification of ten different serotypes can be regarded as a manifestation of the heterogeneous reservoirs and sources of NTS contamination. *S.* Newport was the most prevalent serotype, compared to the more commonly reported *S*. Enteritidis and *S*. Typhimurium in poultry isolates [37–40]. All the NTS serotypes identified in this study are zoonotic, and are known to have caused human disease outbreaks elsewhere. Consequently, this high prevalence of zoonotic NTS in the poultry reservoir constitutes a public health threat. *S.* Typhimurium, *S.* Newport, *S*. Hadar and *S.* Heidelberg have also been reported by Centers for Disease Control and Prevention (CDC) as the most threatening serotypes to public health because of their association with multidrug-resistance [41]. *S*. Mbandaka has been reported in many poultry products across the world [41–45]. A recent publication by Afema et al. [40] reported detection of mainly *S*. Kentucky, *S.* Heidelberg, *S*. Enteritidis, *S*. Typhimurium, and *S*. Virchow from poultry faeces. Also another study by Ikwap et al. [46] reported most of the serotypes of this study in isolates from piggeries in Uganda.

Studies of antibacterial susceptibility in NTS from African countries show highly variable results. An occurrence of almost 58% of isolates resistant to at least one antibiotic, is higher than what was reported in a similar study in Chad, that found overall resistance to 16 antibiotics tested at 33% [13]. However, it is lower compared to a study in Sudan that reported antibiotic resistance in NTS isolates at 98% [47] and similar to a more recent one in Ghana that reported resistance at 60.6% [48]. In Ethiopia, a study on NTS isolates from dairy cattle by Eguale et al. [49] found resistance at 30%. This high level of resistance could be associated with overuse and misuse of antibiotics in poultry farming. The significantly higher resistance level of NTS from Wakiso, which is the immediate district surrounding the capital Kampala should be of concern. Kampala is the biggest hub of trade and movements of people, animals and animal products in the country, and resistant bacteria can potentially spread from here to all regions in the country.

Multidrug-resistance was seen in *S*. Bolton, *S*. Mbandaka, *S*. Hadar, and *S*. Newport isolates. High levels of multidrug-resistance have been reported elsewhere in Africa [11, 12, 48, 50]. The bacteria expressing resistance towards antimicrobials of which some are commonly used in humans and animals exposes a daunting challenge. Increasing development of antimicrobial resistance against commonly used drugs like ciprofloxacin, tetracyclines, sulphonamides sulfamethoxazole_trimethoprim (co-trimoxazole) in Uganda poises a great threat to public health and economy. Tetracyclines and sulphonamides are among the most widely used drugs for treatment and prophylaxis in food animals [51]. Increasing resistance toward these antimicrobials will render them less available leaving farmers with no cheaper options. In Uganda, ciprofloxacin is not licensed for use in poultry production, but is widely used for treatment of many human infections, including salmonellosis. The mechanisms behind the observed high resistance to ciprofloxacin in this study needs to be investigated.

Resistance to sulfamethoxazole-trimethoprim (co-trimoxazole) was also seen in this study. Co-trimoxazole is a drug that is used in Uganda for controlling opportunistic infections in persons living with HIV/AIDS [52, 53]. This causes concern as many of these patients will succumb to opportunistic infections [52–54] and also considering that most patients cannot afford other options of antimicrobials. All isolates were susceptible to the extended-spectrum beta-lactams (meropenem, cefotaxime, ceftazidime) that were tested. Efforts should be put in place to maintain this status. Strategies to reduce antimicrobial resistance in Ugandan farm settings should focus on improving management, biosecurity, and sensitization of key stakeholders such as farmers, farm workers, policy makers, drug dealers, animal health workers and veterinarians.

At univariate analysis, a number of demographic farm management and production variables were associated with occurrence of NTS on farms. The final logistic regression model built identified large farm size, presence of other poultry species, and keeping of records as factors associated with NTS in Uganda. The final model was also tested for the random effect of village to assess the degree of independence. While initial analyses indicated that village had some effect, the final model revealed no such effect – indicating that the factors found were stable across the study districts. A large farm was one of the risk factors associated with NTS determined by the model. This is in concordance with the fact that farm size has been significantly associated with presence of NTS in studies conducted in Britain [26], Trinidad and Tobago, Grenada, and St. Lucia [55], France [15, 56] and Belgium [57]. A previous study of presence of NTS in layer and broiler flocks in the Kampala region in Uganda by Nasinyama et al. [58] identified bird type, flock size and downtime as significant risk factors. In the Ugandan setting, bigger farms tend to attract many activities; visitors and the obtaining of feeds, feed ingredients, chickens and other supplies from multiple sources, many of which informal and unregulated. Under these situations with low biosecurity practices, keeping adequate hygiene standards can be difficult. In addition, most routine operations like mixing of feeds, feeding of birds, watering and vaccinations are manual requiring many workers, some of them coming from outside the farms. Some farms have a limited workforce and are therefore less effective in keeping high standards of routine hygienic practices. All these factors can be further complicated by farms experiencing erratic outage of power and water supplies. As a range of management factors are related to farm size, we may not have identified the most biologically important causal factors.

The current study identified presence of other animal species as another risk factor for NTS. When present on farms, other animal species will most likely share water feeds and space with the chicken and thereby increasing the opportunities for the spread of the bacteria due to direct or indirect contacts. The other animal species may be reservoirs of NTS and thus, contribute to the maintenance of high prevalence of NTS at a farm.

Surprisingly, keeping records emerged as a factor increasing the risk of infection. This could be because in Uganda record keeping is poor among smallholder farmers, and this study found a significant association of NTS with large farms. Keeping records is therefore probably correlating with large farms. It remains open if this variable should be in the model, but leaving out this variable did not affect the estimates for the other variable much. The variable is therefore not considered to be a confounder, but it may perhaps represent other factors.

Having separate housing for birds newly introduced in the farm was associated with lower levels of NTS. The lower level of NTS in farms using separate houses for new chicken is as expected and may represent other factors related to general hygiene, potentially reducing the risk of introduction and maintenance of NTS at farms. Normally housing new birds separately provides an opportunity to observe and provide timely treatment before they are released to mix with other birds on the farms.

The prevalence estimate in the current study is associated with several uncertainties. The study was a cross-sectional study including only one sampling occasion per farm. Depending on the infection levels, it is possible that in some farms a small proportion of birds shed NTS in faeces and this is normally intermittent [59]. Consequently, the prevalence and number of NTS in faecal droppings may therefore change over time and analysis of this requires a longitudinal monitoring scheme. The sampling of faecal droppings only, in order to determine the presence or absence of NTS, may be a limiting factor as NTS in farm settings can be carried in litter, feeds and water as well. However, pooled faecal and environmental sampling of poultry houses is still better than sampling individual birds for detection of NTS on farms as reported in previous studies [60, 61]. In addition, this study targeted commercial egg laying farms who are registered, and yet a lot of farmers are not registered with NAADS. However, these results provide an important insight into the occurrence of NTS in Ugandan poultry, particularly in the absence of previous similar studies. In this study, data on the sources of day old chicks were not captured and this could be included in future studies of NTS in Uganda as sources of day old chicks are potential risk factors for *Salmonella.* In addition, the sampling strategies limited identification of more than one serotype in small farms with only one house as only one sample was taken and one colony was picked for serotyping.

## Conclusions

A high prevalence and high levels of antimicrobial resistant NTS in commercial laying chicken farms in Uganda was revealed in this study. Large farms and presence of other animal species at the farm were identified as risk factors for NTS. Both these risk factors are associated with biosecurity challenges. Although limited, this study should pave way for informing the establishment of proper NTS control systems based on empirical scientific evidence.

Further characterization of NTS from the poultry reservoir that is documented through the present work will be necessary in order to elucidate the transmission dynamics and dissemination of these important zoonotic bacteria. Particular emphasis needs to be given to the determination of antimicrobial resistance genes and their mobility in future studies.

## Additional file

Additional file 1: Questionnaire used for study data collection. (DOCX 33 kb)

## Abbreviations

CDC: Centre for Disease Control and Prevention
CI: Confidence interval
MDR: Multi-drug resistance
MSRV: Modified Semi solid Rappaport Vassiliadis (MSRV)
NAADS: National Agricultural Advisory Services
NTS: Non-typhoidal *Salmonella*
UNCST: Uganda National Council of Science and Technology
WHO: World Health Organization
XLD: Xylose Lysine Deoxycholate
