# Supplementary material for: Prevalence, antimicrobial susceptibility and risk factors associated with non-typhoidal Salmonella on Ugandan layer hen farms
Source: BMC Vet Res. 2017 Nov 29;13:365. doi: 10.1186/s12917-017-1291-1 (PMC5707898; doi:10.1186/s12917-017-1291-1)
Supplement: Additional file 1: — Questionnaire used for study data collection. (DOCX 33 kb) [file 12917_2017_1291_MOESM1_ESM.docx]

Dear …………………………………………….

**PHD RESEARCH QUESTIONNAIRE**

My name is ____________________________ and I am working on behalf of Makerere University College of Veterinary Medicine Animal Resources and Bio security. This questionnaire is part of a PhD research of Dr Terence Odoch Amoki (+256 772 360168) that is being undertaken at Norwegian University of Life Science, through a collaborative research project. The questionnaire will collect data on poultry production practices, diseases, prevention, control and treatment. In addition fecal sample from poultry houses maybe taken you’re your farm. Information collected will be used for research purposes only and high level of confidentiality will be observed.

If you permit, I would like to ask you a few questions. Do I have your permission to continue?

**Interview Date**: __________________

***Identification number***

**A. PERSONAL INFORMATION OF THE FARMER/FARM MANAGER/ATTENDANT**

A.1. Name of the farmer

A.2. Location of the farm GPS Position. . ………………………………….

A.2.1. Village (LC 1)……………….… A.2.2. Parish ………………..

A.2.3. Sub-County…………………… A.2.4. District………………………

A.3. Sex of farmer M F

A.4. Education level of the farmer

(1) None (2) Primary (3) Secondary (4) Tertiary (Specify main area)…………..

A.5. Education Level of the farm manager/attendant

(1) None (2) Primary (3) Secondary (4) Tertiary (Specify main area)

A.6 Gender of the farm manager/attendant:

(1) Male (2) Female

A.7 Age (in years) of the farm manager/attendant

(1) <20 (2) 20 – 35 (3) 36 – 50 (4) >50

A.8 Any other occupation of the farmer: ……………………………………..

**B. POULTRY MANAGEMENT PRACTICES *(You may tick more than one choice where necessary)***

B.1. What species of Livestock do you keep?...............................................

B.2. Do you have pets (1) Yes (2) No If yes go to B3 if no skip B3

B.3. What pets do you have (1) Dogs (2) Cats (3) Both dogs and Cats (4) Others (specify)…………..

B.4. What is the size of your poultry farm?

(1) Small: 50 – 500 birds (2) Medium: 501 - 1000 birds (3) Large >1000

B.5. What poultry species are kept in this farm/home?......................

(1) Ducks (2) Turkeys (3) Pigeon (3) Guinea Fowls (4) Others (Specify)……..

B.6. What are the main breeds of chicken kept on the farm

(1) Local-native (2) Exotic (Layers) (3) Exotic (Broilers) (4) Mixed (Local and exotic)

(5) Exotic Layers and Exotic Broilers) (6) Others…………..

B.7. What is the age of the current flock (in weeks)? (Multiple answers allowed)

(1) Less than 8 weeks (2) 9 – 20 weeks (3) 21 – 45 weeks (4) More than 45 weeks

B.8. How many poultry houses do you have?................................................

B.9. What is the capacity of each of the poultry house? (multiple answers allowed).....................................................

B.10. Do you keep birds of different ages at the farm (1) Yes (2) No

If yes go to B11, if no skip B12

B.11. Do you keep birds of different ages in the same house? (1) Yes (2) No

B.12. Where do you obtain your poultry?

(1) Gift (2) Buy from market (3) Hatchery (4) Dealers of day old chicks (Agents) (5) Others (Specify)………….

B.13. What type of poultry management do you apply

(1) Free range (2) semi- intensive (3) intensive (4) Others (Specify)…………….

B.14. If management is intensive or semi intensive, who is the main person who takes care of the poultry?

1. Husband (2) Wife (3) Children (4) Relative (5) Employee (6) Others (specify)………….

B.15. Do you practice ***all in all out*** system of management? (1) Yes (2) No

B.16. What is the type of housing for your poultry?

(1) Kitchen (2) Chicken house (3) Main House (3) Garage (5) Others (specify)……

B.17. Do your poultry share housing with other birds, animals or humans? (1) Yes (2) No

If yes go to B18, if no skip B18

B.18. If yes, which species

………………………………………………………………………………………………

B.19. Do your poultry intermingle or mix freely with birds/ animals in the neighborhood? (1) Yes (2) No

B.20. Do you restrict persons entering the poultry house? (1) Yes (2) No

B.21. If yes, what type of restrictions? …………………………………………….

B.22. Do you control rodents and flies in your poultry house?

(1) Yes (2) No If yes, go to B.22 and B.23, if no go to B24

B.23. What do you do to control rodents?......................................................

B.24. What do you do to control flies?.........................................................................................

B.25. Have ever seen a rodent on this farm in the last 6 months? (1) Yes (2) No

B.26. What is your main source of feeds?

(1) Ready to use commercial feeds (2 ) Home mixed rations (3) Kitchen wastes

(4 ) Mill bye products (brans etc.) (5) Whole grains (maize, millet sorghum etc)

(6) Others (specify).... . . . . . . . . . . . . . . . .

B.27 Where do you buy the feeds or feed ingredients from?..................................

B.28 How often do you replace litter?

B.29. What is your main water source (sources)?

(1)Family tap water (2) Public tap water (3) Bore hole (4) Open well (5) Surface water (spring, pond, river)

(6) Others (specify)…………………….

B.30. How long have you been in poultry farming? (1) less than 1 year (2) 1 – 5yrs (3) More than 5 yrs.

B.31. Do you keep records on this farm? (1) Yes (2) No

B. 32. What types of records do you keep?
 (1) Treatment records (2) Vaccination records (3) Disease records (4) Production records (5) Sales records

(6) Others (Specify)……………………………….

**C. POULTRY DISEASES PREVENTION MEASURES**

C.1. Do you have a written biosecurity plan (1) Yes (2) No

C.2. Do you apply measures to prevent diseases in your poultry? (1) Yes (2) No

If yes, list the measures (eg. Quarantine, cleaning, disinfection, vaccination)

..................................................................

C.3. Do you use disinfectants in your poultry house? (1) Yes (2) No (If yes go to C3)

C.4. What type of disinfectants? (May show you the containers)

………………………………………………………………………….

……………………………………………………………………

C.5. How often do you disinfect your poultry house?

………………………………………………………..

- How do you carry disinfection?

......................................................

C.6. Do you clean and disinfect between flock? (1) Yes (2) No

C.7 Do you provide footbath at the entry of each poultry house (1) Yes (2) No

C.8. Do you have a disinfectant for vehicles entering your farm (1) Yes (2) No

C.9.Do you have dedicated personnel clothing and equipment for poultry production? (1) Yes (2) No

C.10. Do you put on protective clothing (Gumboots, overalls etc.) while in the poultry house? (1) Yes (2) No

C.11. Do you have a separate hand washing facilities for farm use (1) Yes (2) No

C.12. What is your down time (time between clearing the house and bringing new stock)?

C.13. Do you use egg trays (1) Yes (2) No

C.14. Do you reuse egg trays? (1) Yes (2) No

C.15. How do you clean egg trays?..............................................................

………………………………………………………………………………..

C.16. Do you vaccinate your poultry? (1) Yes (2) No

If yes, go to C 11 if no go to C11

C.17. Who does the vaccination?

1. Government/Local Government worker (2) Non- Governmental organization worker (3) Private provider

(4) Neighbor (4) Family member (5) Others (specify)……………….

C.19. What is the source / supplier of the vaccines

(1) Government (2) Non- Governmental organization (3) Private sector

C.20. What diseases do you vaccinate your poultry against?

1. New Castle Disease (2) Gumboro (3) Fowl Typhoid (4) Fowl pox (5) Mareks disease (6) Infectious Laryngitis

(7) Others (Specify)

C.21. Do you have a vaccination schedule that you follow? (verify by seeing it) (1) Yes (2) No

C.22. Do you have a separate poultry house for new birds? (1) Yes (2) No

C.23. Do you have a separate poultry house for sick birds? (1) Yes (2) No

C.24. Do you sometimes see the presence of wildlife/wild bird present in areas where your birds are

housed (1) Yes (2) No

C.25. Do other animals share water sources with your poultry? (1) Yes (2) No

**D. POULTRY DISEASES AND MANAGEMENT**

D.1. Do you get poultry diseases on this farm? (1) Yes (2) No If yes go to D2,

D.2. What are the diseases do you get on your farm ?

……………………………………………………………………………………………………………………………………………..........................................................................................................................................................................................

D.3. What disease signs do you normally see? (eg Cough, diarrhea, loss of weight, blood in feces, discharges from nose, eyes)

…………………………………………………………………………………………………………………………………………………………………………………………………………………………………………………………………………………………………………………………………………………………………………………………………………

D.4. When disease occurs, which is the most affected age group?

(1) Chicks (Less than 4 weeks) (2) Growers/Pullets

(3) Adults (4) All age groups

D.5. What do you do when birds get sick?

(1) Eat (2) sell off (3) Isolate them from healthy birds (4) Seek diagnosis/ treatment

(5) Others (specify)

D.6. If you treat your poultry, who mainly does the treatment?

(1) Self with traditional medicine

(2) Self with modern medicine

(3) Government Veterinary/ Animal health worker

(4) Private Veterinary/ Animal health worker (5) NGO Veterinary/ Animal health worker

(6) Community Animal Health worker (

(7) Traditional medicine man (woman)

D.7. What is the main outcome of this treatment?

(1) Recovered (2) Died (3) Still sick (4) Others (Specify)

D.8. What is the extent of losses/deaths over one year?

(1) None (2) moderate (less than 50%) (3) Severe (above 50%)

D.9. What do you do when birds die?

(1) Dispose off (2) Eat (3) Others (Specify)…………………..

D.10. What are the sources of your drugs supplies?

(1) Government ( 2) Non- Governmental organization 3) Private sector Vet/Agrovet Drug shop

D.11. How do you dispose dead birds?

…………………………………………………

D.12. Do you access animal health extension services?

(1) Yes (2) No

D.13. If yes, who provides?

(1) Government (2) Non- Governmental organization (3) Private

D.14. Who mainly pays for this service?

(1) Government (2) Non- Governmental organization (3) Self

**THANKS**
